# Referral care for high-risk pregnant women in rural Rajasthan, India: a qualitative analysis of barriers and facilitators

**DOI:** 10.1186/s12884-022-04601-6

**Published:** 2022-04-11

**Authors:** Saachi Dalal, Ruchit Nagar, Rohaan Hegde, Surya Vaishnav, Hamid Abdullah, Jennifer Kasper

**Affiliations:** 1 Warren Alpert Medical School of Brown University, Providence, USA; 2 Khushi Baby, Udaipur, India; 3 Department of Internal Medicine and Pediatrics, Yale New Haven Hospital, Yale University, New Haven, USA; 4 Imperial College London, London, UK; 5 Department of Global Health and Social Medicine, Brigham and Women’s Hospital, Department of Pediatrics, Harvard Medical School, Boston, USA

**Keywords:** Maternal health, Referral care, High risk pregnancy, Rural health, Barriers, Facilitators, Community health workers, India

## Abstract

**Objective:**

To qualitatively assess the barriers and facilitators to uptake of referral services amongst high-risk pregnant women in rural Rajasthan.

**Methods:**

A purposive sample of pregnant women with high-risk conditions requiring referral follow-up care (severe hypertension, moderate anemia, and severe anemia) were considered for inclusion. In-depth individual interviews were conducted in the local dialect, Mewari. Interviews were transcribed, coded, and organized for thematic generation as per the analytical framework described in the socio-ecological model.

**Results:**

19 high risk pregnant women of low socioeconomic backgrounds across 15 villages were interviewed. Barriers to referral care included lack of transportation, household responsibilities, and limited awareness, education, and social support. The most prominent barrier was lack of accompaniment to the referral center by a family member or health worker. Facilitators included available husbands, engaged heath workers, supportive neighbors, and other female family members who shared past experiences.

**Conclusions:**

Social support at the interpersonal and community level was key to overcoming referral care barriers faced by high-risk pregnant women in rural Rajasthan. Interventions that enhance social support may improve uptake of referral care services by high-risk pregnant women.

## Background

In India, an estimated 30,000 mothers die annually from preventable causes related to pregnancy and childbirth [1]. Meanwhile, 800,000 children under the age of five die from vaccine preventable disease, neonatal infection, birth asphyxia and malnutrition. In 2017, the Indian Council of Medical Research (ICMR), Public Health Foundation of India (PHFI) and National Institute of Nutrition (NIN), reported malnutrition as the main risk factor for under-5 deaths nationally, accounting for 68% of total deaths. A major predictor of low birthweight and infant malnutrition is maternal anemia (and associated poor maternal nutrition), which occurs in greater than 50% of women between the ages of 15–49 years [2].

Rajasthan, India’s largest state, is a high-focus state with respect to reproductive and child health (RCH). It has a population of 80 million citizens [3], 75% of whom live in rural areas [4], and a maternal and infant mortality rate of 199 (India MMR: 130) and 41 (India IMR: 34) respectively [5, 6]. Annually, an estimated 420,000 pregnant women in Rajasthan are at high-risk of delivery complications from carrying three or more pregnancies[7].

**Fig. 1:**
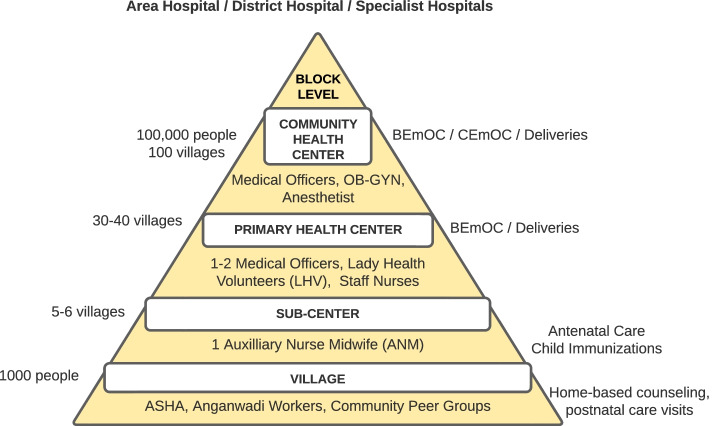
In India, the public health system begins at the village level, where the ASHA (accredited social health activist) spreads health awareness for multiple health programs, identifies and counsels eligible couples for family planning, guides pregnant women to referral facilities, and identifies postnatal infant danger signs. At the sub-center, the ANM (auxiliary nurse midwife) conducts antenatal care and child checkups, provides nutritional supplements, and performs immunizations. Here, women and children are identified as high risk and referred for higher level care at the primary health center, where the Medical Officer (MO) treats them and conducts deliveries

India’s public health system (Fig. 1) [8] provides a large set of cost effective, successful solutions across the ante-, intra-, and post-natal care continuum that have been implemented to prevent avoidable maternal, neonatal, and child mortality: iron folic acid supplementation for pregnant women, regular antenatal care checkups, institutional deliveries, training on newborn care, immunizations, and treatment of febrile illness [9].

However, since data for these interventions is collected on paper at the point of care, the public health system faces the following problems: missing and inconsistent data, poor coordination between health workers at different levels of care, lack of accountability, delays in reporting data for action, unregistered populations, and a disconnected continuum of care [10, 11].

To address these gaps, Khushi Baby, a non-profit organization based in the Udaipur District of Rajasthan, developed and implemented a digital health intervention for community health workers to track key reproductive and child health indicators over the last five years. The system also includes field staff who support community health workers by facilitating referral care visits for high risk beneficiaries. So far, 15 field staff have conducted more than 2000 home and hospital visits to encourage completion of referral care for maternal anemia and infant malnutrition. Overall, the Khushi Baby system has been shown to improve data completeness, data consistency, infant immunization rates and infant malnutrition [12].

In spite of these interventions, high-risk pregnant women and infants in rural Udaipur still receive inadequate referral care services, motivating the objective of this study – to investigate the barriers and facilitators to uptake of referral services amongst high-risk pregnant women in rural Rajasthan.

As described by Thaddeus and Maine, mothers face three key delays to referral care: delays in seeking care, delays in arriving at the healthcare facility, and delays in provision of adequate care [13]. These delays are rooted in socio-economic, cultural, and environmental characteristics (patient factors) and quality of health care (health system factors). *Health system* factors are thought to carry more weight than *patient *factors because of their potential to affect all three phases of delay [13].

A 2019 narrative review of barriers of maternal and neonatal referral systems in developing countries by Harahap et al. noted challenges in both patient and health systems factors. Patient barriers included: environments, knowledge about the referral, poverty, maternal health status, and culture. Health system barriers included: transportation, communication, quality of care, referral documentation, standard procedures for referral and monitoring, and network infrastructure. The five studies from India (between 2014–2018) included in the review focused more on the health system and did not identify barriers related to network infrastructure, knowledge about referral, maternal health status, and culture [14].

Barriers to maternal and child referral care have been under-researched in the Indian context, and most relevant for our study, no such studies have been conducted in Rajasthan. We hypothesize that beyond the globally identified barriers to maternal and child referral care, several specific barriers may emerge from a local application of the socio-ecological model.

The objective of this study is to conduct qualitative interviews with high risk pregnant (HRP) women living in rural communities in Udaipur to 1) identify barriers and facilitators to referral care completion, 2) categorize them into individual, interpersonal, community and structural factors, 3) study the interactions and combined effects of individual/interpersonal factors and community/structural factors on referral care completion, and 5) discuss potential policy, human resource and technology solutions to improve referral care completion and improve maternal and infant health. To our knowledge, this study is the first to use the socio-ecological model (SEM) to qualitatively assess the barriers and facilitators of antenatal referral care in India.

## Methods

### Selection and recruitment of participants

Pregnant women suffering from moderate to severe anemia (Hb < 10) and/or hypertension (BP > 140/90) were identified as high risk by the Khushi Baby digital health mobile application (data collection tool at government health camps) and eligible for our study. Using purposive sampling, recruitment and interviews were conducted with participants from four geographical blocks: Gogunda, Sarada, Salumbar, and Jhadol. These participants were selected to represent varying geographical terrains, community values and referral completion rates. Participants were recruited from these blocks until data saturation had been achieved for the majority of themes [15]. We restricted our selection process to these four blocks because of long term relationships between Khushi Baby field monitors and community members living in these areas. Khushi Baby field monitors selected participants and organized interviews but were not personally involved in the interview process. Only individuals who provided verbal consent and felt able to complete the interview were recruited.

### Data collection

In-depth interviews were conducted in January 2021 in Mewari, the local dialect, by a native of Udaupir and experienced public health researcher, SV. All interviews were conducted in participants’ homes and lasted 30–45 min. Several participants performed the interview with their infants present due to unavailability of child care. Although it was advised against, some relatives or neighbors supported the participants in their personal responses. In some cases, we requested a trusted member of the community, such as the ASHA, to be present to provide support or additional information regarding community values and societal resources. Participants were told they could decline to answer any questions and could stop the interview at any time.

The interviews followed a semi-structured interview guide which included open-ended questions on personal background, socio-economic status, past experiences with public health services and personal and community attitudes towards pregnancy, antenatal care and family planning.

During the in-depth interviews, verbal responses were translated by the interviewer and transcribed by one of the researchers. Participant transcripts and surveys were labelled with participant ID numbers in order to maintain confidentiality in the reported data. All files were password protected and stored on a secure computer network with restricted access.

### Data analysis

Summary notes informed the preliminary analysis of participant transcripts. In developing an analytic thematic framework, data was initially indexed and categorized using an inductive approach. To further identify relevant themes in our study, a subsample of transcripts (< 20%) were studied by four members of our research team: Saachi Dalal (BA, MD and MSc Population Medicine candidate), Ruchit Nagar (BA,MPH,MD), Rohaan Hegde (BA), Hamid Abdullah (MSW, Field Implementation Lead at Khushi Baby). The reviewers assigned codes to specific responses, constructed thematic maps and compared themes that showed a significant level of consistency. Through discussion, prominent categories were refined, new categories were identified and discrepancies were resolved. Data was summarized using thematic matrices [15] and a socio-ecological model (SEM) was used to organize relevant themes and present results. The model selected for our study was inspired by the SEM as conceived by McLeroy, et al. [16, 17]. Noteworthy narratives and quotes were also selected to encapsulate themes and their interactions.

## Results

We completed interviews with 19 participants. Sociodemographic details are shown in Table 1 below. We report individual and interpersonal barriers and facilitators such as personal and family awareness, beliefs and attitudes, and social support in Tables 2 and 3 below. We then present factors at the community and structural level such as opportunities for peer learning and access to public transportation in Tables 4 and 5 below. The relative importance of factors which influence referral completion are illustrated in the discussion section.

**Table 1:**
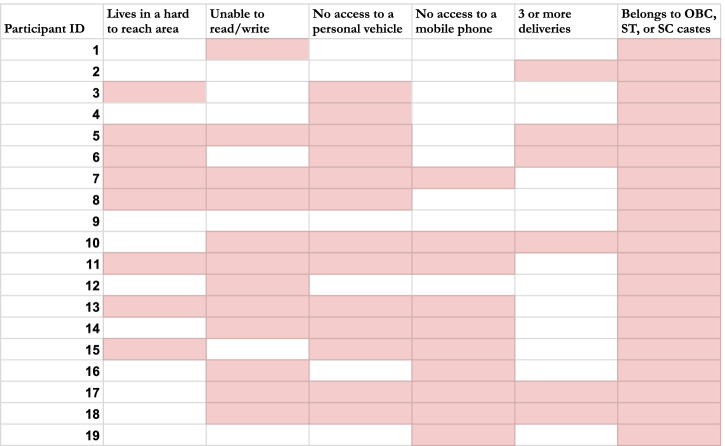
Socioeconomic factors at an individual level among the study population

**Table 2:** Individual and Interpersonal Barriers

Factor	Quote	Barrier / Facilitator
Level of Education	“I need my father-in-law to take me to the referral center because he is the only one in our family who speaks well and understands the doctor. My husband is not smart enough to take me and I cannot go alone.” “My brothers went to school, but my parents did not send my sisters and I to school.” “My father did not work and used to drink alcohol, so my older sister and I did not attend school. We had to take care of our younger siblings and help with housework. We also looked after the goats and took on small jobs around the village to earn money.” “My siblings and I only attended school till fourth or fifth grade because we had to take care of the goats, work in the field, and help around the house.”	A lack of or low level of education was identified as a barrier to completing referral care. We observed that issues with literacy and communication hampered the beneficiary’s ability to travel independently, navigate the health facility, provide information to health staff, and comprehend medical explanations and advice. A majority of participants were illiterate due to a lack of or minimal formal education for the following reasons: gender bias, child marriage, prioritization of work over school due to poverty or paternal absence, and negligence towards education.
Low Health Awareness	“The ANM sister has told me I have anemia and that I need to go to the hospital to get medicine and a bottle (IV medicine). I have gone to the hospital once before when my stomach was hurting badly, but I did not go this time because I only experience dizziness sometimes. I am feeling okay.”	Majority of women were identified as having low awareness about their health condition, associated symptoms, effects on maternal and newborn health at delivery, and treatment options. The decision to seek care is directly related to an ability to understand and weigh the risks and benefits of living with the health condition versus receiving medical treatment at the referral facility. Many women who did not complete their referral were aware of symptoms and treatment options, but almost all of them were unaware of possible delivery complications resulting from their high-risk conditions.
Low Health System Awareness	“I have never been to the hospital, so I do not know what happens there. There is a bus stop near my house, but I do not know how to reach the hospital, so I did not go. My husband will come back from his labor work in Udaipur next month. I will wait for him to take me. I will not go alone.” “I know the ambulance comes to our village. But when I went into labor, I did not know the ambulance phone number, so we chose to call a private jeep to take us to the hospital.”	Knowledge about preventative and referral care services, where and how to access them, and health staff familiarity provide ability and comfort in navigating the referral care process. For example, several women were not comfortable going to the referral care facility on their own due to lack of understanding the process and system. Many were also unaware of the ambulance phone number and specific government designated health service days, when the ASHA or ANM accompanies pregnant women from the village to the referral center for antenatal care.
Personal/Family Attitudes and Beliefs	“The ANM comes and goes. She doesn’t care about me and my family like Dai Ma (local midwife) does. Dai Ma takes care of my children when I am busy and stays with me during my delivery and after.” “What do you mean by ‘male sterilization’?! This cannot happen.” “My husband cannot do the sterilization procedure because he will not be able to work if he does it. So, I will do it.” “This is my fifth pregnancy… I wanted to have the sterilization surgery after my second child, but since my husband was the only boy amongst his siblings and we only have one son, my father in law wants us to keep trying until we have a second male child. So what could I do? I have to obey my elders.”	Several participants displayed personal and cultural beliefs that directly influenced their decision to seek referral care. For example, a few participants, despite high education and awareness, displayed negligence towards the ANM’s advice regarding referral care, and only visited the PHC when experiencing severe symptoms. Others avoided seeking referral care due to fear of the unknown or lack of trust in the government public health system. Some mothers from nomadic communities have more faith in local healers and midwives. Often, decisions towards family planning are influenced by the beliefs and attitudes of paternal family members, e.g. husband, mother-in-law, and father-in-law. Several participants whose personal preferences lined up with those recommended by the ANM often were compelled to make contrary decisions based on their family’s wishes.
Lack of Access to Personal Transport	“To get to the block hospital, my husband and I had to walk for 30 min just to get to the bus station. After a sonography, the doctor told us I needed to be given a bottle (IV Iron-Sucrose), but the journey and wait time was so long that it had become evening. We were worried about catching the bus to get back home so we left without treatment. So, the doctor gave us two IV bottles to take home. The next day, we had to travel to the ANM’s house, where she administered the medicine.” “A month ago, I felt labor pains in the night and my husband used his mobile phone to call a private vehicle. However, by the time the car arrived an hour later, my baby had been born. My mother-in-law and husband were there during the process and helped cut the umbilical cord.”	Lack of access to personal transport, such as a motorbike or a car, was identified as a significant barrier for women living in areas with poor access to public transport. Many women reside in remote areas with difficult terrain located 1 to 5 km away from the nearest local bus stop, warranting 20 to 60-min walks. Few women lacked access to public transport, and had to cover the entire journey to the referral center on foot. Since the majority of participants either lacked trust or knowledge regarding the ambulance system, access to personalized transport became especially relevant for labor and delivery. All participants who had home deliveries lacked personal modes of transport, whether owned or borrowed from neighbors.
Lack of Accompaniment to the Referral Visit	“I understand that I am anemic and that my baby will be weak if I do not get treated. If I want to go to the hospital, I can take the autorickshaw, which stops right outside my house and my neighbors will watch my child while I am gone. But my husband works in Udaipur and there is nobody at home to take me to the hospital.”	Many participants identified the main barrier to completing their referral care visits to lack of accompaniment during the process. Accompaniment involved not only assistance with transport and public health system navigation, but also emotional support. Since the primary occupation of families living in rural Udaipur is agriculture or labor, lack of accompaniment to the referral center emerged most for women whose husbands were migrant or day laborers in Udaipur city or other states. In several cases, participants directly attributed failure to complete their referral visit to their husbands being unavailable to take them to the facility. In these cases, participants either did not have family or neighbors willing to accompany them or preferred their husband’s accompaniment.
Household/Financial Responsibilities	“Initially, I did not go to the referral facility because I had to plant tomatoes and take care of the goats. Then, my mother-in-law went to her parents’ house, so the workload at home and in the field increased even more. I had to wait 3 months for her to come back, so that my father-in-law and I could go to the referral facility.”	Majority of women have household responsibilities such as cooking, cleaning, child care and walking long distances to acquire drinking water for the family. Additionally, many have financial responsibilities such as agriculture and animal husbandry. Because most women are not comfortable going to the referral facility on their own, often the process involves more than one family member neglecting their daily responsibilities. Many participants identified this issue as the reason for delays in their seeking referral care.

**Table 3:** Individual and Interpersonal Facilitators

Social Support	“The hospital is an hour walk from my home. For most referral visits, I walk with my sister-in-law, but for my last delivery, our neighbors let us borrow their bike so that we could reach the facility on time.” “My husband stays in Udaipur City, where he is a laborer. He only returns once every month, so he cannot take me to the hospital. Usually, my sister-in-law or neighbor walks with me to the nearest bus stop and we go together. Once, my neighbor took me and his pregnant wife, who also was due for a referral, on a motorbike.” “I do not have my own mobile phone but I have my husband’s mobile number printed on a wall in my house. He lives and works in Udaipur City, so if I need to call him my neighbors let me borrow their mobile phone.” “Our family does not have a mobile phone, but we use our neighbor’s mobile phone to call a private car or an ambulance when we have to go to the hospital for delivery.” “If I need to go to the hospital with my husband, my sisters-in-law or neighbors take care of my children and watch our house.”	For the participants who overcame these barriers and completed a referral visit, the strongest facilitator was social support from extended family or neighbors in the following forms: access to a personal vehicle for transport, accompaniment to the referral visit, access to a mobile phone for communication, and household support such as childcare.

**Table 4:** Community and Structural Barriers

Poor Access to Public Transport	“It takes one hour to walk to the closest primary health center. There is no bus or auto which can take me there and my husband is too busy with work to take me on our family motorcycle.” “I usually go to the primary health center by bus. It is a long and tiring journey because I have to walk for 30–40 min to reach the bus stop.”	Some participants experienced significant delays in the referral process because public transport was not accessible. The government bus routes only operate from Udaipur City to the local block hubs and are effectively unserviceable to families traveling from local villages to the primary health center. Therefore, referral visits can only be completed through arrangement of a personal vehicle, privately operated buses or auto-rickshaws, and the public ambulance. Additionally, public infrastructure (e.g. roads or dirt tracks), is not always present, preventing access to buses, auto-rickshaws and ambulances. In some areas, despite the presence of roads, participants still have to cover 3—4 km on foot to reach the nearest, privately operated, bus stop. Some participants living in geographically isolated areas reported a complete lack of access to transport. Without access to personal transport vehicles, the majority of these pregnant women could only reach the primary health center by walking for 45 to 60 min. This lengthy journey can take all day resulting in the woman arriving home by late evening. Pregnant women find it difficult to accomplish the journey alone due to safety reasons, exhaustion, financial or household responsibilities, child care, lack of education/awareness, and lack of a mobile phone for communication.
Poor Service at Primary Health Center	“I have gone to the block hospital two times for my anemia. Both times, I walked by myself for one hour to reach the bus station. After which, the bus journey takes around thirty minutes. At the hospital I waited two hours before the doctor could see me. The first time I went, I was given medicine. The second time, I was told to return back home because the doctor was not available. Since then, I have not gone back to the hospital.” “The ANM sister referred me to the block hospital for my anemia. So, I went to the hospital to pick up my iron tablets. But at the hospital the doctor didn't give me any supplements. He told me I needed to get a sonography done at a private clinic, and that he would only give me the tablets once I had returned with the sonography. I wanted to do my sonography, but my mother in law fell ill for a few months and I had to take on extra responsibilities at home. I have missed my last few referrals because I still haven't got my sonography done.”	At the referral facilities, the two significant barriers experienced by some of the participants were the unavailability/absence of doctors and insufficient medical aid provided. Many of the participants were deterred from completing their subsequent referrals due to negative experiences at the PHC. When doctors were not present at the facility, participants were either treated by a nurse, or were asked to come back on a later date. For those couples that travel long distances and sacrifice their household/financial responsibilities in order to complete their referrals, being asked to return the next day presents a significant burden In some cases, failure to provide the necessary medications as well as poor bedside manner by PHC medical professionals can also act as deterrents to future referrals. In one such rare case, the doctor at the PHC withheld the required medications until the patient completed specific referral procedures at private clinics.
Lack of ASHA support	“The ASHA does not visit any of the houses in our village or accompany us to the referral center. She doesn't even call or visit to remind us to go to the referral center or the Anganwadi for our antenatal care visits.” “I am of the Gameti caste, which is a backward caste in my village. That is why the ASHA does not visit my house.” “My baby was delivered at home one month ago without any help from the ASHA or Dai Ma (SBA). The ASHA or ANM have not come to visit me or my child yet, so my child has not been vaccinated.”	One of the primary responsibilities of the village ASHA is to ensure that pregnant women complete their referral visits, either through a household visit/phone call reminder or by accompanying the women to the referral facility as a travel companion and patient care navigator. The ASHA must also ensure women are aware of and have access to reliable modes of transport to reach the hospital for an institutional delivery. If a woman prefers to have a home delivery, the ASHA must ensure that a skilled birth attendant (SBA) is present. After the child is born, the ASHA must visit the household seven times during the first 42 days to identify infant danger signs as part of the government ‘Home Based Neonatal Care’ (HBNC) program. A majority of the participants stated that ASHA neither reminded them to go to the referral facility nor accompanied them. Almost all of the participants who had children under the age of 5 stated that the ASHA did not visit their new born child for HBNC visits. Our experience has shown us that ASHA’s perform poorly primarily due to excessive workload or negligence. There are some villages where the culture of caste discrimination affects delivery of health services as well.
Geographic Isolation	“I have had six deliveries of which five have been home deliveries. I was initially scared of going to the block hospital, but after going once I realized it was alright. For my most recent delivery I was planning on going to the hospital, but I went into labor in the middle of the night and I was alone. So, I delivered my child on my own and used an old blade to cut the umbilical cord.”	In few cases the homes of participants were located in geographically isolated areas, separated from nearby villages by a few kilometers. The women living in these areas primarily experience barriers to transport as they have to walk significant distances, of 2 -5 km, in order to access public roads and public transport. The inaccessibility of these locations often results in a high rate of home deliveries, sometimes without any assistance. Additionally, without many neighbours, the participants that lived in isolated areas lacked basic awareness of the health system as well as the social support required to seek out and complete referral care.
Lack of Community Peer Groups	“I do not know of any community meetings for pregnant women in the village. I do meet other pregnant women at the Anganwadi center when I go to receive my antenatal care services at the monthly health camp. But there is no community discussion that happens on the camp day.” “I know there are *Samuhas* (self-help groups) in my village but I do not attend because I don’t know much about it and I don’t have time.” “I used to attend the Samuhs in my village but they were discontinued because not many women joined. I talked to my husband and my friends about my pregnancy, but the discussions at the Samoh were mostly about money.”	Several government programs such as Village Health and Sanitation Committee, Village Health and Nutrition Camp, and Adolescent Health include a provision for community meetings for women within reproductive age to promote peer learning. However, most participants stated that they were not aware of any community spaces designated for them to gather with other pregnant women to talk, either freely or through a facilitated discussion with a government health worker. Many participants were aware of Self-Help Groups (Samuh) aimed to improve financial literacy and independence among women. Although these groups could also serve as spaces for women from marginalized communities to socialize or discuss reproductive and child health issues, most participants did not attend or stated that the discussions were usually only related to finances.
Lack of Confidence in the Ambulance System:	“I did not call the ambulance because I went into labor in the night. We thought the ambulance does not come at night so we called a private jeep, but by the time it had arrived, the baby had already been born at home.” “When I went into labor, we called the ambulance. They said they were coming, they said they were coming! We waited for a while, but when the ambulance did not come, we hired a local jeep for Rs. 600 to go to the hospital… It is expensive for us, but what else could we have done?”	The barriers associated with the ambulance system was a general lack of knowledge or trust. Families were aware of financial incentives associated with institutional delivery but did not know how to call the ambulance, doubted its reliability, or held misconceptions that it is not functional at night. All participants, except one, did not use the ambulance to reach the hospital for delivery. These participants paid high prices to rent a private vehicle at the time of labor, borrowed a neighbor’s vehicle or delivered at home. Lastly, there were rare cases of ambulances not making it to the participant's homes in time.
Lack of Access to Referral Documentation	“I am in my 6th month of pregnancy, but the ANM sister has not yet given me my Mamta Card. She writes in it and keeps it with her at the Anganwadi center. She has told me to go to the referral center but I have not gone because I need to show my MAMTA Card there. I am scared that if I do not show it, I will be sent back home without treatment. Next month when I go to the health camp, I will get the MAMTA card from the ANM sister. After that I will go for my referral treatment.”	The Mamta Card is a paper based health card provided to beneficiaries to track their journey during pregnancy, delivery and early childhood development and immunization. The Mamta Card serves as documentation during the referral process to ensure informed care at every level. It is also used as evidence of successful care completion, which is later linked to financial incentives for health workers and beneficiaries. However, it is often not filled out, because the ANM lacks the time to fill both the Mamta Card and her paper register during the health camps. Many participants mentioned that the ANM keeps the Mamta Card with her until the last month of pregnancy. The ANM keeps the Mamta Card to fill out after the health camp is over when she has time. She also keeps it to ensure the beneficiary does not lose it as it holds important information linked to incentives. This has left beneficiaries without the resources to 1) understand their health status and pregnancy timeline and 2) present their medical history at the referral center.

**Table 5:** Community and Structural Facilitators

Availability of Public Transport	“My husband lives in Udaipur, so I am here alone most of the time. I have supportive neighbors who care for my children when I need help, but I usually don’t have anyone to accompany me to the hospital. I have been told by the ANM sister to visit the hospital during my pregnancy. So far, I have gone 3 times. I usually take the bus that comes to a stop 5 min away from my house. I do not know much about my health, but when I went to the hospital, I was given a bottle (IV Iron Sucrose).”	A majority of the participants displayed a strong reliance and trust in privately operated bus or auto transport as a means to reach the referral facilities. Since a large number of participants lacked access to personal vehicles, the accessible public modes of transport were observed to be strong facilitators for completing referrals, medical emergencies as well as deliveries.
ANM/ASHA Support	“My ASHA always comes to my house to remind me about health camps. When I had to go to the hospital, she accompanied me because I did not have anyone at home to take me. We walked for one hour to reach the hospital where I was given a bottle (IV Iron Sucrose).”	The village ASHA’s notable responsibilities include visiting beneficiaries houses to deliver health awareness messages and health camp reminders, conduct home based neonatal care to identify infant danger signs, and accompany pregnant women to their referral care visits and deliveries at higher level health centers. The latter provisions have been established to support mothers in travel, health system navigation and emotional distress. Several participants with low awareness or a lack of social support appreciated how their village ASHA would visit their homes regularly and accompany them to the primary health center for their antenatal care referral visits or deliveries.

## Discussion

This qualitative study among high-risk pregnant women in rural Udaipur identified barriers and facilitators to completing referral care at both an individual/interpersonal level and a community/structural level in rural Rajasthan, India. Although the presence and relative importance of factors varied for each participant, our findings reveal a broader understanding of referral care experience among this population.

In comparison to conclusions of Harahap et al.’s narrative review of barriers of maternal and neonatal referral systems in developing countries, this study found patient factors to be of greater relevance than health system factors for successful completion of referral care. [14] Specifically, the most prominent facilitator of referral care was identified as social support, specifically by a husband, family member or the ASHA for travel and care navigation. Even women with no additional barriers at the individual or structural level failed to complete referrals without accompaniment. Outliers who completed referrals without accompaniment had one or more prior deliveries, and access to child-care support and convenient transportation options nearby. Unlike the three delays model which identifies quality of the public health system as the leading factor for referral completion, our analysis showed that the patient’s connectedness to a long-term accompagnateur was more relevant. Similarly, other research in India has found that the degree of social support, especially by husbands and ASHAs, significantly influences prenatal health behaviors, institutional delivery, and health outcomes for pregnant women [18, 19].

At a village level, ASHAs have been recruited to fill gaps of low health awareness and social support through counseling, accompaniment, and facilitation of health seeking behaviors. ASHAs are financially incentivized to ensure pregnant women receive at least one antenatal care checkup, to accompany them during delivery, and to complete seven home visits for post-natal screening. Notably, ASHAs are responsible for but not financially incentivized to motivate pregnant women to complete advanced antenatal screening and high-risk referral care. All participants, except two, stated that ASHAs did not visit their home regularly to deliver reminders and awareness messages, did not accompany them to the referral center for high-risk care or deliveries, and did not screen newborns for danger signs.

Lack of initiative by ASHAs in Udaipur may be associated with being overburdened and underpaid by the government health system. ASHAs in Rajasthan, despite having equal responsibility, receive a lower base salary compared to ASHAs in other states. They are expected to make up the difference through task-based incentives; however, they do not receive support or financial incentives for facilitation of referrals. Additionally, poor communication and coordination between ASHAs and MOs at the primary health center leaves ASHAs without information regarding which beneficiaries have missed their referral care visit. Finally, based on our field observations over many years, poor monitoring from higher level authorities leads to ASHAs falsely reporting tasks as completed for the sake of receiving incentives. Ultimately, poor accountability, capacity building, support and incentives for ASHAs negatively affects the beneficiaries they serve.

Studies based in Pakistan and Bangladesh have found concordance with our findings, citing similar key barriers to referral completion: limited coordination between community health workers and the referral facility, lack of incentives or support to community health workers in facilitating the referral and lack of peer groups for social support [20, 21]. Studies in Bangladesh also emphasized the following facilitators in direct association with our findings: 1) community engagement and self-help groups to develop action plans, promote awareness and provide support; 2) reimbursement schemes or community funding linked to referral care support, and 3) community health workers accompanying the patient to the referral facility [21, 22].

Finally, beyond social connectedness and lack of ASHA support, reasonable access to transportation was identified as essential for referral care completion and safe delivery. Since none of the villages are served by government buses, many participants who otherwise go independently to the village health camp (within a 1 km distance), required accompaniment to reach the referral center. If public transport were easily accessible, more women who have health system awareness may be comfortable completing referral care visits on their own.

This study had certain limitations, specifically many participants gave limited responses due to the personal nature of the interview, despite having a female interviewer speaking in their native dialect. These interviews were not electronically recorded, which limited the number and accuracy of quotes captured.

The strengths of the study come from the data-driven recruitment approach. The prior rapport with field monitors and familiarity with a female voice in the local dialect likely strengthened the response quality. The study captured a diversity of sociodemographic conditions by interviewing women over a 100 km + radius around Udaipur.

## Future directions

Future studies may explore the perspectives of caregivers, health workers, and government officials to gain a holistic picture at both an individual and structural level. Retrospective studies comparing delivery outcomes with antenatal history of such social determinants may corroborate study findings. Further, the following solutions are proposed to account for the specific barriers and facilitators identified in the study, in the context of the local Khushi Baby intervention and resources:

### Digital health census

To improve ASHA engagement, the Khushi Baby team is developing a digital health census mobile application. Through this platform, we plan to screen for social connectedness (e.g. history of participation in a peer learning group, rating of ASHA engagement during pregnancy and postnatal care, number of days husband is away from home each month, number of adult female family members living in the house, or access to family member or trusted neighbor for accompaniment during health-related emergencies or referral visits) and structural issues (e.g. distance to PHC, access to and types of transportation, knowledge about ambulances) to assist with health system navigation.

### High risk beneficiary prioritization

Khushi Baby’s mobile application for the ANM has generated a database of more than 20,000 pregnant women to date, of which more than 8,000 women have delivery outcome and infant health data. We will use these details to develop a high-risk score for each beneficiary. From these scores, a list of prioritized high-risk beneficiaries with their degree of their social connectedness, distance to referral facility and due dates, will be displayed for the ASHA and ANM on their mobile applications to inform them which women require extra support during pregnancy and delivery. Additionally, this technology has potential to increase communication and collaboration between ASHAs and ANMs.

### Health worker coordination

ASHAs may use this tool to keep running list of home visits and ensure they follow-up with beneficiaries according to program due dates (e.g. deliveries and post-partum maternal and newborn care). The tool will also provide reminders for peer-learning sessions mandated through health policies. To support ASHAs in improving their health communication skills, locally-tailored audiovisual materials to counsel mothers regarding their need for referral care will also be included. To motivate ASHAs to use the tool and complete their responsibilities, the application will automate monthly reports and display the expected monthly incentives payout. It is envisioned that this automated tool will financially empower ASHAs and overcome dependency on data entry operators to report their work to ultimately receive compensation for key services provided at the village level. Accountability mechanisms in the form of data quality checks, GPS tracking and biometric authentication will be integrated into the app to confirm that ASHAs are actually visiting households and interacting with beneficiaries. Altogether, the aim of this application is to digitally empower ASHAs to focus on providing high-quality care in their respective villages, particularly to high-risk beneficiaries.

**Fig. 2:**
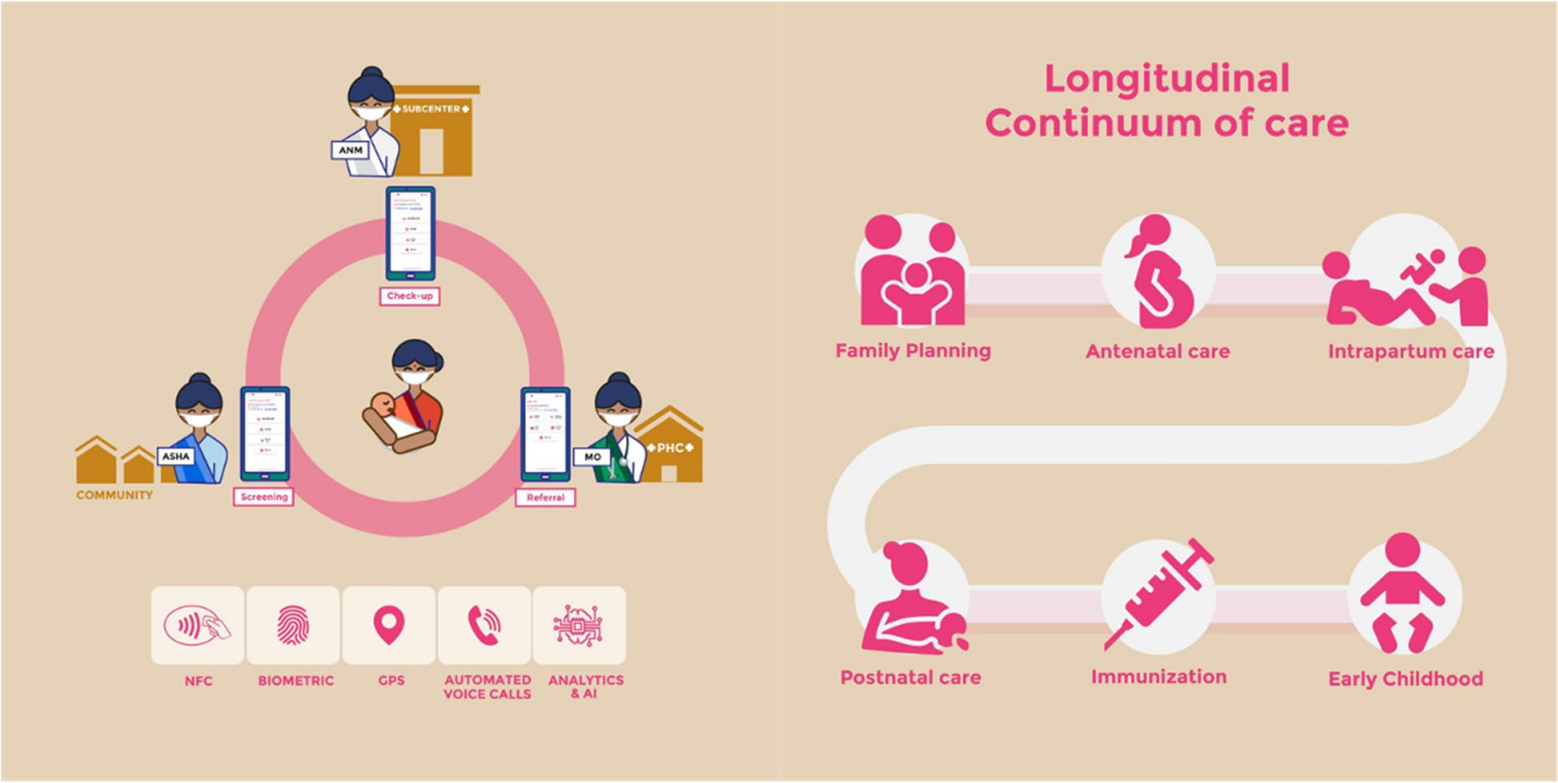
Khushi Baby RCH continuum of care: mobile application for ASHA, ANM, MO, automated voice calls for beneficiaries, NFC, GPS and biometric authentication, and dashboard analytics and AI

To complete the chain of referral (Fig. 2), Khushi Baby is developing a mobile application for Medical Officers at the primary health center. Beyond other functions, this app will sync with the ASHA and ANM apps so that all three groups of health care providers (ASHAs, ANMs, MOs) will work from the same list of high-risk beneficiaries due for referral, see who did not complete their referrals and call them through the app to investigate and communicate reminders.

### Targeted and automated communication

Additionally, Khushi Baby currently sends automated voice calls to the family to complete antenatal care visits and infant immunizations. This system has been very helpful to ASHAs who travel across tough terrain to deliver these reminders door-to-door. Findings from this study will inform new content to specifically remind and motivate pregnant women to complete referrals. We may also refine our voice call content to target fathers, who account for the majority of mobile phone owners, and to address misconceptions regarding ambulance availability. Further, introducing SMS and interactive voice response system messages (IVRS) to gauge whether the respondent understood or found the content useful can also be integrated to evaluate their effectiveness over time. Finally, Khushi Baby call center will make targeted personal call to pregnant women due to deliver in the coming week to discuss and establish a safe delivery plan, including social accompaniment and transportation.

### Policy reform

At a larger scale in Rajasthan, policy decisions will need to be made to supercharge potential facilitators to referral care. We recommend that the Department of Medical, Health, and Family Welfare consider an incentive model tied with an accountability mechanism for high-risk pregnant women to avail the Pradhan Mandiri Surakshit Matritva Abhiyan scheme, which provides free advanced antenatal care screenings for pregnant women on the 9th of each month. The Department should also consider how to improve capacity building and financial support for ASHAs who are instrumental to carrying out these referrals through accompaniment. Information, education and communication campaigns may be targeted to close specific awareness gaps at the village level, particularly regarding schemes and availability of specific ambulance support for pregnant women. Leaders of village-level self-help-groups should be motivated and supported to facilitate discussions around women’s health issues during scheduled meetings on loan disbursements. Finally, husbands should be engaged through directed village meetings to discuss the process and importance of referral visits and safe delivery plans. Multiple strategies, addressing each layer of the socio-ecological model, will need to be implemented in a coordinated manner to improve uptake of high-risk pregnancy referral care and ultimately better maternal and child health outcomes in Rajasthan, India.

## Conclusions

In rural Udaipur, Rajasthan, high risk pregnant women face numerous barriers to seeking referral care which include lack of accompaniment to the health center, limited education and health awareness, lack of transportation, geographic isolation, and household/financial responsibilities. However, social support at the interpersonal and community level has been shown to overcome barriers—accompaniment to the referral center was identified as the strongest facilitator to completing referral care.

## Data Availability

Anonymized quotes have been listed in the results section above. Full transcripts are not available for review because they have personally identifying information. Redacted transcripts may be requested from the corresponding author upon reasonable request.

## Abbreviations

ANC: Antenatal care checkup
ANM: Auxiliary Nurse Midwife
ASHA: Accredited Social Health Activist
BeMONC: Basic emergency obstetric and newborn care
CeMONC: Critical emergency obstetric and newborn care
CHC: Community health center
DMHFW: Department of Medical, Health, and Family Welfare, Government of Rajasthan
Early initiation of breastfeeding: Provision of colostrum to the child
Full antenatal care completion: 4 ANCs, tetanus injection, 180 Iron Folic Acid tablets received
Full infant immunization: Completion of PENTA 1–3, OPV 1–3, Measles, and BCG immunization
HRP: High risk pregnancy
IFA: Iron folic acid tablets
IMR: Infant mortality rate
IVRS: Interactive voice response system
Minimum acceptable diet: As defined per National Family Health Survey guidelines
MOIC: Medical Officer in Charge
MMR: Maternal mortality rate
OBC: Other backward caste
PHC: Primary health center
PMSMA: Pradhan Mandiri Surakshit Matritva Abhiyan scheme
RCH: Reproductive and child health
RMNCH + A: Reproductive, maternal, neonatal, child, and adolescent health
Safe delivery: Delivery in the presence of a certified skilled-birth attendant
SC: Scheduled caste
SHC: Sub-health center
ST: Scheduled tribe
VHND: Village health and nutrition day camp
